# Clinical significance of cumulative biological effective dose and overall treatment time in the treatment of carcinoma cervix

**DOI:** 10.4103/0971-6203.33244

**Published:** 2007

**Authors:** Abhijit Mandal, Anupam Kumar Asthana, Lalit Mohon Aggarwal

**Affiliations:** Department of Radiotherapy and Radiation Medicine, Institute of Medical Sciences, Banaras Hindu University, Varanasi, UP, India

**Keywords:** Biologic effective dose, carcinoma cervix, radiotherapy

## Abstract

The purpose of this retrospective study is to report the radiotherapy treatment response of, and complications in, patients with cervical cancer on the basis of cumulative biologic effective dose (BED) and overall treatment time (OTT).

Sixty-four (stage II - 35/64; stage III - 29/64) patients of cervical cancer were treated with combination of external beam radiotherapy (EBRT) and low dose rate intracavitary brachytherapy (ICBT). The cumulative BED was calculated at Point A (BED_10_); and bladder, rectal reference points (BED_2,5_) using the linear-quadratic BED equations.

The local control (LC) rate and 5-year disease-free survival (DFS) rate in patients of stage II were comparable for BED_10_ <84.5 and BED_10_ >84.5 but were much higher for BED_10_ >84.5 than BED_10_ <84.5 (*P*< 0.01) in stage III patients. In the stage II patients, The LC rate and 5-year DFS rate were comparable for OTT <50 days and for OTT >50 days but were much higher in stage III patients with OTT < 50 than OTT >50 days (*P*< 0.001). It was also observed that patients who received BED_2.5_ <105 had lesser rectal (*P*< 0.001) and bladder complications than BED_2.5_ >105. Higher rectal complication-free survival (CFS_R_) rate, bladder complication-free survival (CFS_B_) rate and all-type late complication-free survival rate were observed in patients who received BED_2.5_ < 105 than BED_2.5_ >105.

A balanced, optimal and justified radiotherapy treatment schedule to deliver higher BED_10_ (>84.5) and lower BED_2.5_ (< 105) in lesser OTT (< 50 days) is essential in carcinoma cervix to expect a better treatment outcome in all respects.

Radiation therapy is the standard treatment of choice in all stages of cancer of the uterine cervix, with the combination of external beam radiotherapy (EBRT) and intracavitary brachytherapy (ICBT).

Radiotherapy is delivered through different types of treatment units with variable physical parameters (e.g., target conformity, type of radiation, dose rate, etc.). Large number of treatment schedules (fractionation scheme, dose of EBRT and ICBT, time gap between EBRT and ICBT, etc.) are available. Sometimes planned treatment may change from original due to unavoidable circumstances. Therefore, these variations during treatment should be unified by radiobiological parameters such as biological effective dose (BED)for late-reacting tissue (BED_2.5_), for acute-reacting tissue (BED_10_) and overall treatment time (OTT). The radiobiological evaluations are helpful to clinicians in evaluating the various treatment schedules quantitatively to predict the treatment outcome.[1–3]

The aim of this retrospective study is to report our critical radiobiological assessment of patients with cervical cancer treated in the year 1997. The predictive quality of radiobiological parameters in treatment outcome is also analyzed.

## Materials and Methods

This study highlights the radiobiological analysis of 64 patients of cervical cancer treated in one unit of radiotherapy in the year 1997. Following criteria were used to analyze the patients. Previously untreated patients with histopathology of squamous cell carcinoma were only included in this study. All cases were clinically staged as per FIGO classification and were treated by radical radiotherapy, which essentially includes both EBRT and ICBT. ICBT applications were performed in accordance with ICRU-38[4] by remote after loading (RAL) Selectron unit or by manual after loading (MAL) Amersham unit. Out of 64 patients, 54.69% were in stage IIB, and 45.31% were in stage IIIB. Age of patients ranged between 27 and 65 years, with a median of 45.5 years [Table 1].

**Table 1:** Patient demography

Total number of patients	64
Follow-up (median, range)	37 months, 19-90 months
StageII (No, %)	35 (54.69%)
StageIII (No, %)	29 (45.31%)
Age (median, range)	45.5 years, 27-65 years
Calculated BED_10_ (median, range)	84.53, 72.67-89.71
Calculated BED_2.5_ (median, range)	108.44, 73.19-126.19

### Treatment plan and schedule

#### External beam radiotherapy

Majority of the patients received 4400 cGy (range 4,000-4,500 cGy) in 22 fractions (range 20-22 fractions) with daily fraction of 200 cGy (range 200-204 cGy) in 30 days (range 27-36 days) by external beam radiotherapy (Co-60) to the pelvis prior to the intracavitary brachytherapy insertion. Patients with interportal distance (IPD) <20 cm were treated by two parallel opposed AP/PA portals (89%), and those with IPD ≥20 cm were treated by ‘four field box’ technique (11%) with two AP/PA and two lateral portals.

Intracavitary application: Time gap of 14 days was given between completion of external beam radiotherapy and brachytherapy application. Both RAL Selectron unit and MAL Amersham unit were equally used for the intracavitary brachytherapy applications.

#### Amersham application

Thirty-four patients received ICBT by Caesium-137 MAL Amersham unit; the dose distribution was similar to the Manchester system, and dose rate was 46-47 cGy per hour to point A. Patients received a total brachytherapy dose of 3,200 cGy (range 2,673-3,351 cGy) to point A in 69 h (range 58-71 h). The Amersham system uses permanently loaded flexible-source pencils in combination with standard packs of disposable plastic applicators.

Selectron application: Thirty patients received ICBT by RAL Selectron units. Dose rate was 139.81-145.38 cGy per hour to point A. Because of expected increase in normal tissue complications with increased dose rate, the total brachytherapy dose was reduced by 17.5% as compared to the MAL Amersham dose. Total dose received by point A was 2,640 cGy (range 2,478-2,879 cGy) in 18.88 h (range 17.39-20.29 h). Standard Selectron rigid applicators were used.

#### Dose calculation and treatment planning

Dummy radiographic markers were inserted into intrauterine tandem and ovoids for source localization. Rectal catheter with steel balls having diameter of 2.5 mm were used to locate rectum; and to visualize the bladder, 7 cc of contrast media was put in Foley's catheter. Simulation film of orthogonal film of pelvis with applicators in position was obtained for dosimetric purpose. The rectal and bladder points were taken as specified in the ICRU-38.[4] On the anterior view of the radiograph, the bladder point was taken at the center of the balloon. In the lateral view the reference point was chosen on an anterior-posterior (A/P) line drawn through Foley's balloon center at the posterior surface. The rectal reference point was delineated on a lateral radiograph on an A/P line drawn through the middle of the intra-vaginal source and 5 mm behind the posterior vaginal wall. TSG RAD-PLAN treatment planning system was used for external beam radiotherapy and intracavitary treatment planning. Dose to point A, B, rectal and bladder was obtained. In vivo rectal dosimetry was also carried out.

#### Radiobiological aspects

The BEDs were calculated at point A (BED_10_); and bladder, rectal reference points (BED_2.5_) using the linear-quadratic BED equations.[3] The equations of BEDs in fractionated and continuous treatments are as given bellow - [A] and [B]:

$${\mathrm{BED}}_{\mathrm{fr}}=\mathrm{Nd}\left[1+\frac{d}{\alpha /\beta }\right]\;\mathrm{(A)}$$

N = number of fractions

d = dose per fractions (Gy)

α/β = tissue-specific parameter (Gy)

$${\mathrm{BED}}_{\mathrm{cont}}=\mathrm{RT}\left[1+\frac{2R\left(1-1/µT\right)}{µ\left(\alpha /\beta \right)}\right]\;\mathrm{(B)}$$

R = dose rate (Gy/hr)

T = application time (hr)

µ = tissue-specific parameter

α/β values were taken as 2.5 Gy for normal tissue late effects and 10 Gy for tumors.[3] μ values were taken as 0.46 hr^−1^ for normal tissue late effects and 1.40 hr^−1^ for tumors.[3] The cumulative BED_10_ and BED_2.5_ were calculated by addition of contribution of external beam radiotherapy and low dose rate brachytherapy. The median of calculated cumulative BED_10_ and BED_2.5_ was 84.53 (range 72.67-89.71) and 108.44 (range 73.19-126.19) respectively.

#### Follow-up

After completion of treatment, all the patients were put on routine follow-up. Patients were evaluated for local disease control and any evidence of distant failure. Rectal and bladder complications were also evaluated throughout follow-up period. The late rectal and bladder toxicity were graded according to the Radiation Therapy Oncology Group (RTOG) criteria. The follow-up ranged between 19 and 90 months, with a median of 37 months for all patients.

Statistics: All data was statistically analyzed using Statistical Package for Social Sciences (SPSS), version 10.0. Survival curves were derived using Kaplan-Meier cumulative survival method, and significance of the data was tested using Chi-square test.

### Results

#### Local control

Out of 35 patients of carcinoma of cervix stage II, 17 received BED_10_ < 84.5 and 18 received BED_10_ >84.5. The local control rate was 76.5 and 77.8% for BED_10_ <84.5 and BED_10_ >84.5 respectively [Table 2]. Sixteen patients received the full course of radiotherapy in <50 days and 19 in >50 days. The local control rate was 75.0 and 79.0% for <50 days and >50 days respectively [Table 3].

**Table 2:** BED_10_ vs. response and disease-free survival rate

*Stage*	*BED_10_*	*No. of patients*	*L/C %*	*Significance*
II	<84.5	17/35	76.5	Not
	>84.5	18/35	77.8	Significant
III	<84.5	14/29	78.6	*P*<0.01
	>84.5	15/29	93.4	Significant

**Table 3:** Overall treatment time *vs.* response and disease-free survival rate

*Stage*	*Overall treatment time*	*No. of patients*	*L/C %*	*Significance*
II	<50	16/35	75.0	Not Significant
	>50	19/35	79.0	
III	<50	12/29	100	*P*<0.001
	>50	17/29	76.5	Significant

Out of 29 patients with carcinoma of cervix stage III, 14 received BED_10_ <84.5 and 15 received BED_10_ >84.5. The local control rate was 78.6 and 93.4% for BED_10_ <84.5 and BED_10_ >84.5 respectively [Table 2]. Twelve patients received the full course of treatment in <50 days and 17 in >50 days. The local control rate was 100 and 76.5% for < 50 days and >50 days respectively [Table 3].

#### Survival

**Figure 1:**
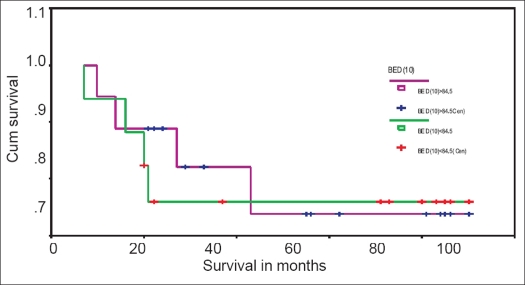
Kaplan-Meyer disease-free survival curve for stage II patients with BED10 >84.5 and BED10 <84.5

**Figure 2:**
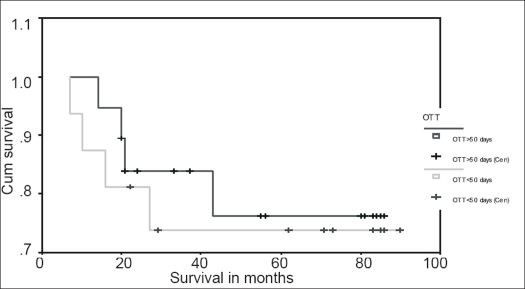
Kaplan-Meyer disease-free survival curve for stage II patients with OTT <50 days and OTT >50 days

In stage II patients, the 5-year disease-free survival (DFS) rate was 76.0% for BED_10_ <84.5 and 73.9% for BED_10_ >84.5 [Figure 1]. The 5-year DFS rate was 73.3% for <50 days and 76.3% for >50 days [Figure 2].

**Figure 3:**
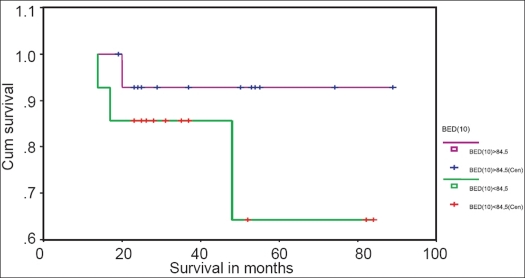
Kaplan-Meyer disease-free survival curve for stage III patients with BED10 >84.5 and BED10 < 84.5

**Figure 4:**
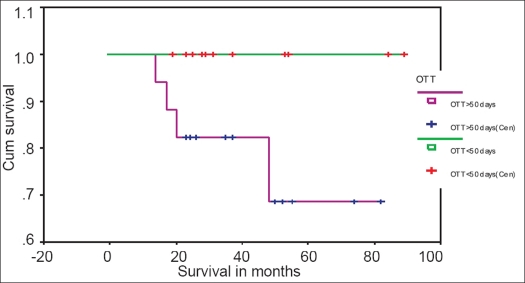
Kaplan-Meyer disease-free survival curve for stage III patients with OTT <50 days and OTT >50 days

In stage III patients, the 5-year DFS rate was 64.3% for BED_10_ <84.5 and 94.0% for BED_10_ >84.5 [Figure 3]. The 5-year DFS rate was 100% for <50 days and 68.6% for >50 days [Figure 4].

#### Normal tissue complications

**Figure 5:**
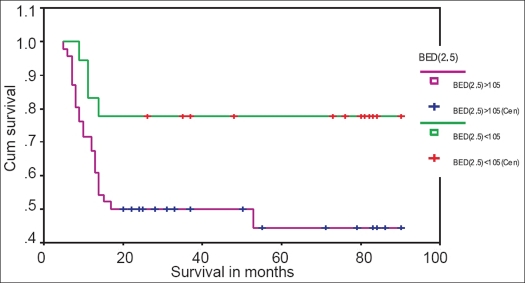
Kaplan-Meyer rectal complication-free survival curve for patients with BED2.5 <105 and BED2.5 >105

**Figure 6:**
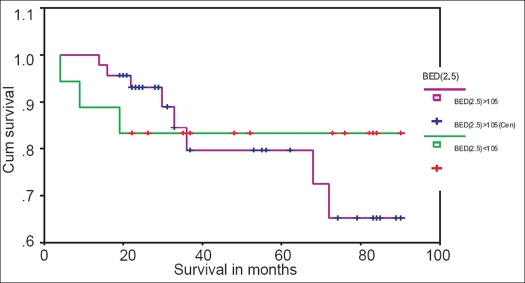
Kaplan-Meyer bladder complication-free survival curve for patients with BED2.5 <105 and BED2.5 >105

**Figure 7:**
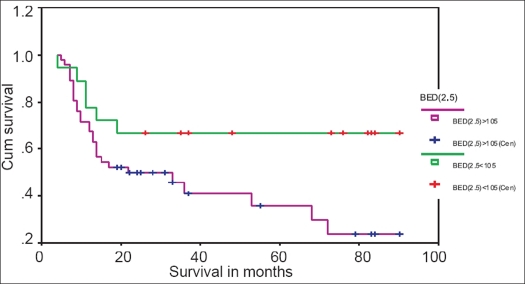
Kaplan-Meyer overall complication-free survival curve for patients with BED2.5 <105 and BED2.5 >105

Out of 64 patients, 18 received BED_2.5_ <105 at rectal and bladder points, and 46 patients received BED_2.5_ >105. Of these 18 patients who received BED_2.5_ <105, 4 (22.2%) had rectal complications (2 patients Gr-II, 1 patient Gr-III, 1 patient Gr-IV) and 3 (16.7%) had bladder complications (1 patient Gr-II, 2 patients Gr-III) [Table 4]. At 90 months, 77.8, 83.3 and 66.7% were the rectal complication-free survival (CFS_R_) rate [Figure 5], bladder complication-free survival (CFS_B_) rate [Figure 6] and all-type late complication-free survival (CFS) rate [Figure 7] respectively.

**Table 4:** BED_2.5_ vs. complication and complication-free survival rate

*Normal tissue*	*BED_2.5_*	*No. of patients*	*Complication*					*No reaction*	*Significance*
					
			*0*	*I*	*II*	*III*	*IV*		
Rectum	<105	18/64	14	0	2	1	1	77.8	*P*<0.001
	>105	46/64	22	7	9	6	2	47.8	Significant
Bladder	<105	18/64	15	0	1	2	0	83.3	Not
	>105	46/64	38	0	4	2	2	82.6	Significant

Out of 46 patients who received BED_2.5_ >105, 24 (52.1%) had rectal complications (7 patients Gr-I, 9 patients Gr-II, 6 patients Gr-III and 2 patients Gr-IV) and 8 (17.4%) had bladder complications (4 patients Gr-II, 2 patients Gr-III and 2 patients Gr-IV) [Table 4]. The CFS_R_ rate [Figure 5], CFS_B_ rate [Figure 6] and CFS rate [Figure 7] at 90 months were 44.4, 65.3 and 23.9% respectively.

## Discussion

External beam radiotherapy and low dose rate intracavitary brachytherapy are widely used for effective management of carcinoma cervix[5] in many centers of developing countries. The low dose rate conventional brachytherapy has added advantages in terms of radiobiology and cost-effectiveness. LQ models predict rise in late effects and reduction in local control as the dose rate increases.[6] The predictive quality of linear quadratic model in treatment of cervical cancer has been discussed in literatures.[7–11] Sood *et al.*[7] reported that cumulative BED_10_ >89 Gy shows better local control rate, and BED_3_ < 100 Gy was associated with negligible late toxicity.

Wang *et al.*[8] made clinical comparison of two ‘linear-quadratic model’-based isoeffect fractionation schemes of high dose rate intracavitary brachytherapy for cervical cancer and reported that the linear-quadratic model correctly predicted the treatment outcome. Jones *et al.*[12] suggested that the concept of BED, in particular, is useful for quantifying the treatment expectations; however, careful interpretation of results is required before clinical decision. Jones *et al.*[13] evaluated the rate of loss of tumor control with extension of treatment time to assess the relative contributions of radiobiological parameters (radiosensitivity, clonogen doubling time, clonogen numbers and fractionation schedule) to such loss. They predicted rates of loss of tumor control produced by an extension in treatment time as 0.9 and 1.1% per day for a hypothetical randomly selected heterogeneous tumor population. Pitfalls in estimating the influence of overall treatment time on local tumor control have been described by Tucker *et al.*[14]

We observed a comparable local control rate (77.8% *vs.* 76.5%) and 5-year disease-free survival rate (73.9% *vs.* 76.0%) in stage II patients who received BED_10_ >84.5 and BED_10_ < 84.5. However, a statistically significant (*P* < 0.01) greater local control rate (93.3% *vs.* 78.6%) and 5-year disease-free survival rate (93.3% *vs.* 64.3%) were observed in the stage III patients who received BED_10_ >84.5 than in those who received BED_10_ <84.5. It has been also noticed that the stage II patients showed comparable local control rate (75.0% *vs.* 79.0%.) and 5-year disease-free survival rate (73.3% *vs.* 76.3 %) with OTT <50 days and OTT >50 days respectively, but stage III patients showed a statistically significant (*P* < 0.001) higher local control rate (100% *vs.* 76.5%) and 5-year disease-free survival rate (100% *vs.* 68.6%) with OTT <50 days than with OTT >50 days respectively. Comparatively poor response in stage II patients compared to stage III patients may be because Selectron rigid application in the stage II patients and flexible Amersham applications in stage III patients are preferably used. The Amersham flexible applicators adapt to the natural anatomy of individual patients, resulting in better dose distribution in target volume; whereas Selectron rigid applicators were molded for the anatomical structure around it, and sometimes dose distribution was compromised.[15] We observed a greater local control and disease-free survival in stage III patients who received BED_10_ >84.5 within 50 days as compared to Sood *et al.,*[7] who found that in 49 patients treated with RT alone, the local failure rate was 10% (1 of 10) and 19% (3 of 16) in patients receiving a BED_10_ >89 Gy_*10*_ or <89 Gy_*10*_ to Point A respectively (*P* = 0.2). They reported local failure rate was 7.7% (1 of 13) and 23% (3 of 13) in patients with a BED_10tf_ >64 Gy_10_ or < 64 Gy_10_ (*P* = 0.1). The expected rise in treatment outcome also compared with other studies. Gasinska *et al.*[10] reported that if OTT exceeded 90 days, loss in BED_10_ for relatively radiosensitive tumors (alpha = 0.3-0.4/Gy and Tk = 28 days) was equal to 0.37-0.26 Gy/day and for radioresistant tumors (alpha = 0.2/Gy), it was 0.6 Gy/day. For fast proliferating tumors (BrdUrdLI >8.8%) BED loss was 1.4 Gy/day and for slowly proliferating tumors (BrdUrdLI ≤ 8.8%), it was 0.2 Gy/day. Kaplan-Meier analysis revealed that OTT ≤ 60 days was a significant prognostic factor for overall survival (OS) (*P* = 0.019), disease-free survival (DFS) (*P* = 0.0173) and local control (LC) (*P* = 0.011). BED_10_ had significant influence on survival (*P* = 0.047).

We have observed a statistically significant (*P* < 0.001) lesser rectal complication rate (22.2% *vs.* 52.1%), higher 90 months rectal complication-free survival rate (77.8% *vs.* 44.4%) and less severe complications in patients who received BED_2.5_ < 105 than in those who received BED_2.5_ >105. Lesser bladder complication rate (16.7% *vs.* 17.4%), higher 90 months bladder complication-free survival rate (83.3% *vs.* 65.3%) and less severe bladder complication were also observed in patients who received BED_2.5_ < 105 than in those who received BED_2.5_ >105. Sood *et al.*[7] reported negligible late toxicity with BED_3_ < 100 Gy_3_. Wang *et al.*[8] reported higher actuarial proctitis rate (49.7% *vs.* 32.7% at 5 years and 50.5% *vs.* 32.7% at 10 years, *P*< 0.001) and higher actuarial cystitis rate (14.3% *vs.* 11.4% at 5 years and 24.1% *vs.* 15% at 10 years, *P* = 0.134) in patients with 146.7 Gy_3_ than in those with 134.4 Gy_3_.

## Conclusion

It has been observed that patients with higher BED_10_ (>84.5) show higher local control rate and disease-free survival rate than the patients with lower BED_10_ (< 84.5) in stage II and stage III. The local control rate and disease-free survival rate are also higher in patients with lower OTT (< 50 days) than in patients with higher OTT (>50 days) in stage II and stage III. Patients with lower BED_2.5_ (<105) had less rectal, bladder complication rate and higher complication-free survival rate than patients with higher BED_2.5_ (>105). Therefore, it can be concluded that to achieve higher tumor control with less normal-tissue complications, BED_10_ should be more than 84.5 and BED_2.5_ should be less than 105 delivered in less than 50 days.
